# Diagnostic Accuracy of Fine Needle Aspiration Cytology for Thyroid Tumours at a Tertiary Hospital in Northern Tanzania

**DOI:** 10.24248/eahrj.v9i2.854

**Published:** 2025-12-24

**Authors:** Bahati Robert, Samwel Chugulu, Kondo Chilonga, David Msuya

**Affiliations:** a Kilimanjaro Christian Medical University College, Kilimanjaro, Tanzania; b Kilimanjaro Christian Medical Centre, Kilimanjaro, Tanzania

## Abstract

**Background:**

Thyroid diseases are common and serious diseases in the world and because of this fact, early diagnosis is necessary. Clinicians in our settings rely on clinical presentation, physical examination and biochemical findings in diagnosing patients with thyroid mass and sometimes this leads to misdiagnosis or late diagnosis of cancerous lesions. Abnormal lymph node sometimes can be mistaken for a thyroid nodule. Clinical examination or biochemical findings become difficult differentiatingdifficult differentiating the two conditions. The global consensus is to employ FNAC as standard baseline test to pick out thyroid lesions with malignancy potential. The inherent pitfalls and unique presentation in developing countries have proven a challenge in using FNAC as a stand-alone tool for preoperative assessment Objective: To determine the diagnostic accuracy of fine needle aspiration cytology for thyroid tumours at a tertiary hospital in Northern Tanzania from January 2019 to June 2023.

**Methodology:**

A retrospective cross sectionalcross-sectional hospital-based study that was conducted from January 2019 to June 2023 in patients who had thyroidectomy done during the study period at KCMC. Structured Questionnaires were used to collect key information from case notes, descriptive data were summarized by median and interquartile range and proportions in percentage and analyzed by SPSS version 25.

**Results:**

A total of 207 patients who underwent thyroidectomy across the period of study were eligible. Females were 89.37% and% and males 10.63% with a Female: Male ratio 8.4:1. The age of participants ranged from 8 to 81 years, median (IQR) 49 (37–56) years. Malignant lesions 47.83% were papillary carcinoma, 30.43% follicular carcinoma and the rest were squamous cell carcinoma 8.7%, follicular%, follicular variant of papillary thyroid carcinoma 8.7% and 4.35% papillary carcinoma follicular variant.

The study had a sensitivity of 36.36%, specificity 91.89%, positive predictive value of 34.78% negative predictive value 92.39% false positive rate 8.10%, false negative rate 63.64% and an overall accuracy of 85.99%.

**Conclusion:**

This study reveals high specificity, a low sensitivity, and a within range diagnostic accuracy for FNAC at detecting malignancy in thyroid nodules. The findings are a baseline data on performance of FNAC in thyroid tumours at our facility, there is a consistent trend toward papillary thyroid carcinoma (PTC) than follicular thyroid carcinoma (FTC) across regions.

## BACKGROUND

Thyroid nodules are detectable in 2 to 6% of the population by palpation and their clinical relevance is because of their malignant potential.^1^ The prevalence of incidental thyroid nodules detected by ultrasonography (US) is estimated to be 20% to 76% in the general population and a 20% to 48% of patients with 1 palpable thyroid nodule are found to have additional nodules when investigated by US.^2,3^ Thyroid nodules are more common in women and older individuals, those living in iodine-deficient areas, in patients diagnosed with Hashimoto's thyroiditis and in people who had been exposed to external irradiation.^5^ Thyroid nodules are a common clinical finding and most are benign, however, 5 to 15% can be malignant.^2,4^

The incidence of thyroid cancer has rapidly increased in the United States and other developed countries over the past 30 years primarily owing to the incidental detection of small-volume papillary carcinomas on imaging studies.^5^ Thyroid cancer (TC) represents about 96% of all endocrine malignancies and one of the most frequent cancers in women and the incidence has increased from about 5 new cases per 100,000 persons observed in the early ’90 to 15 new cases per 100,000 persons recorded in 2012.^6^

The overall incidence of thyroid malignancy has been reported to be 16% in thyroidectomy specimens.^7,8^ Papillary thyroid cancer accounts for 80–85% of diagnosed thyroid cancers, while follicular, medullary, and anaplastic cancers are diagnosed significantly less frequently.^5,4,8^ The ultimate goal when evaluating a patient with a thyroid nodule is to differentiate between benign and malignant lesions or at least to estimate the risk of malignancy in the existing nodule.^3,6,9^ The transformation of endodermal-derived thyroid follicular cells or neural crest–derived thyroid C cells leads to distinct types of cancer.^3,10^

Follicular cells give rise to two main forms of differentiated thyroid cancer: papillary thyroid carcinoma and follicular thyroid carcinoma. Poorly differentiated and anaplastic thyroid carcinomas are comparatively rare tumours of those arising from follicular cells and are associated with aggressive disease.^11,12^ Medullary thyroid carcinoma is the canonical C-cell tumour and has distinct biologic features.^8,13,14^ Iodine deficiency disorders are the commonest cause of thyroid disorders in Africa and the prevalence of endemic goiter ranges from 1 to 90% in different areas of the continent.^15,16,17^

Cystic nodules, representing 10 to 25% of all thyroid nodules, present additional diagnostic challenges and the incidence of malignancy in cysts is probably less than that of solid nodules, nonetheless, complex cystic nodules may be malignant.^18^ The clinical picture and pattern of thyroid disease seen and reported in developed countries may be different from disease pattern in third world countries especially in goiter endemic areas mostly due to challenging socioeconomic status and low literacy levels leading to late presentation with potential for higher malignant degeneration in long standing goiter.^19,20,21,22^ There was a slightslightly higher incidence of malignancy in solitaryin solitary nodule goiter comparedgoiter compared to multinodular goitermultinodular goiter (MNG) patients, though the difference was not statistically significant.

Papillary carcinoma was found to be more common in solitary thyroid nodule (STN) while follicular carcinoma to be more in MNG.^23,24,25^ The most common clinical presentation in the patients who presented with thyroid diseases was neck swelling (Diffuse and Nodular). Other clinical presentations were dysphagia, hoarseness of voice, palpitation, and proptosis.^26^ FNAC of the thyroid gland is now a well-established, first-line diagnostic test for the evaluation of diffuse thyroid lesions as well as of thyroid nodules with the main purpose of confirming benign lesions and thereby, reducing unnecessary surgery.^27,21,28^

This study was conducted to determine the diagnostic accuracy of fine needle aspiration and cytology for thyroid tumours at Kilimanjaro Christian Medical Center (KCMC).

## METHODOLOGY

### Study Design

This was a retrospective cross-sectional hospitalsectional hospital-based study of patients who had thyroidectomy done at KCMC from January 2019 to June 2023.

### Study Area

This study was conducted in surgical department at KCMC. KCMC is a referral hospital based in Moshi Municipal at the foot of Mount Kilimanjaro. It is a referral center with a coverage area of 150,000 square km in the northern part of Tanzania. The population served by KCMC is 15 million people from the local community in the Kilimanjaro region and nearby regions in the northern part of Tanzania such as Tanga, Arusha, and Manyara. Surgical Out Patient Department (SOPD) at KCMC runs its services every Tuesday and Thursday with an average of 50 patients per clinic.

### Study Population

This study includedstudy included all patients who had thyroidectomy done at KCMC from January 2019 to June 2023.

### Eligibility Criteria

#### Inclusion Criteria

All patients post thyroidectomy during the study period.

#### Exclusion Criteria

Patients with incomplete workup.

### Sample Size Estimation

Using Kish Leslie formula (1965)

n = (Z)2P(1-P)/e2

Where n – sample size Z – 1.96

P – Sensitivity of FNAC By (Hassanali et al) = 59.1%,%, accrued based on geopolitical closeness with our region.

e – margin of error = 5%

Therefore, (1.96)20.59(1 – 0.59)/(0.05)2 = 372

A minimum of 372 cases were estimated

### Sampling Technique

Convenience sampling of all patients with FNAC results who underwent thyroidectomies and histopathological results of thyroid lesions between January 2019 and June 2023 at KCMC was conducted.

### Data Collection Methods And and Tools

Case notes retrieval from medical record and extraction of due details on demographic data, cytological findings, histological findings of thyroid lesions using a structured questionnaire were done.

### Data Management and Analysis Plan

#### Data Management Plan

Data were regularly reviewed and audited to ensure its accuracy, consistency, and relevance for data integrity and reliability. Participant data were recorded using codes to maintain privacy and confidentiality of participant information.

#### Data Analysis Plan

Collected data were checked and entered in SPSS version 25 for statistical analysis. Coding and labeling were done according to the variables. Quantitative variables (numerical variables) were summarized into mean, median, range and standard deviation (SD), frequency, and percentage for categorical variables in the form of graphs, frequency tables, and graphs. Sensitivity of FNAC were taken as the ability of FNAC to correctly make diagnoses similar to those made by histological tissue findings.

True positives (TP): All individuals who will test positive for a thyroid disease in both FNAC and histopathology.

True Negatives (TN): All individuals who will test negative for a thyroid disease in both FNAC and Histopathology.

False Positives (FP): Those individuals who will test positive in FNAC but negative on Histopathology.

False Negatives (FN): Those individuals who will test negative in FNAC but positive on Histopathology.

Specificity: is the ability of the test to give negative finding when the tested person is truly free of the disease under study.

Specificity of FNAC will be taken as the ability of FNAC to correctly detect benign lesion in an individual who has a non-reactive/benign lesion by histopathology.

Positive Predictive Value (PVP): Proportion of true diseased individuals amongst all positive tests by FNAC. In other words, signifies the probability that a given patient with positive test, indeed does have the disease.

Negative Predictive Value (NPV): The proportion of healthy individuals amongst all negative tests by FNAC or the probability that a given individual with a negative test indeed does not have the diseases.

Accuracy of FNAC – The proportion of the correct results true positive and true negative in relation to all cases studied. Or process yielding values that are equal on average to the true underlying value for the diagnostic variable measured.

Diagnostic value of the Fine Needle Aspiration Cytology will be checked by calculating the sensitivity, specificity, positive predictive value, negative predictive value and by calculating the percentage of false positive and false negative results.

The 2X2 table was prepared and the following formulae were used to calculate the diagnostic performance:

- Sensitivity = TP/(TP + FN)X100%
- Specificity = TN/(TN + FP)X100%
- PPV = TP/(TP + FP)X100%
- NPV = TN/(TN + FN)X100%
- False positive rate = FP/(FP + TN)X100%
- False negative rate = FN/(FN + TP)X100%
- Accuracy = (TP + TN)/(TP + TN + FP + FN)X100%

The histopathological findings were taken as the gold standard and the results of fine needle aspiration cytology which were matched with the histopathology were taken as positive cases.

The Bethesda System for Reporting Thyroid Cytopathology (BSRTC) was used to group cytological findings

a. Category I: Non-diagnostic or unsatisfactory, malignancy, malignancy potential 1 to 4%; b. Category II: Benign,, malignancy potential −0 to 3%; c. Category III: AtypiaIdiotypic of undetermined significance or follicular lesion of undetermined significance (AUS/FLUS), malignancy potential −5 to 15%; d. Category IV: Follicular IV: Follicular neoplasm or suspicious for a follicular neoplasm (FN/SFN), malignancy potential, 15 to 30%; e. Category V: Suspicious V: Suspicious for malignancy (SUSP) SUSP), malignancy potential, 60 potential, 60 to 75%; f. Category VI: Malignant VI: Malignant, malignancy potential, 97 to 99%

Lesions with Categories IV through VI Bethesda on cytology were regarded as malignant for purposes of analysis given the relatively higher potentials for having a malignant lesion malignant lesion on respective specimens.

Analyses were done by Statistical Package for Social Sciences version 25, P-values less than or equal to 0.05 were considered significant.

### Ethical Consideration

Ethical clearance PG63/2022 was obtained from KCMUCo Kilimanjaro Christian Medical University College Ethics Committee where Waiver for consent was granted. A permission for conducting research was obtained from the KCMC administration.

## RESULTS

**FIGURE 1:**
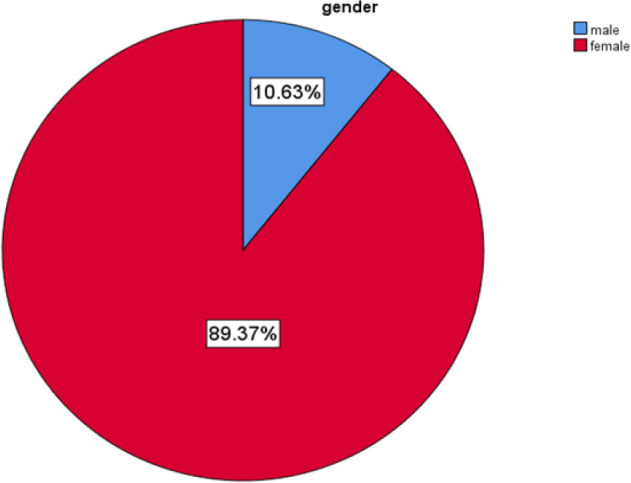
Sex Distribution of Study Population

A total of 217 patients who underwent thyroidectomy across the period of study had their records reviewed for information relevant for the study. Ten patients were found to have incomplete details and were excluded from analysis. That left a total of 207 subjects for analysis. Response rate was 95.4%. Sex categorization had females at 89.37% and% and males 10.63% with a Female: Male ratio of 8.4:1, as depicted on Figure 1.

**FIGURE 2:**
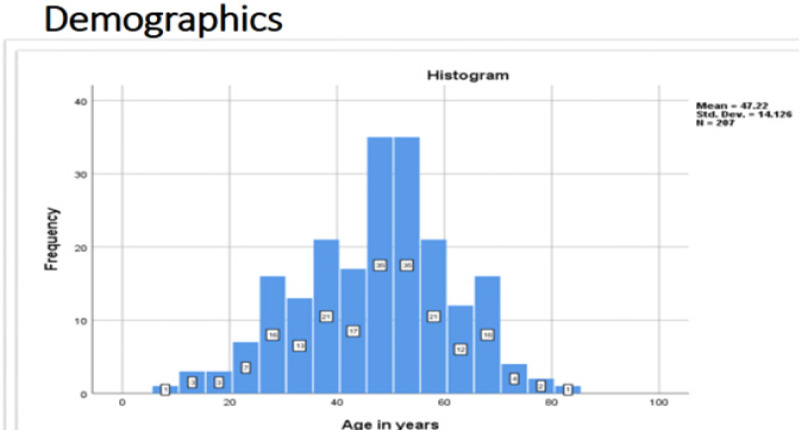
Age Distribution of Study Participants

The age of participants ranged from 8 to 81 years, median (IQR) 49 (37–56) years, as portrayed in Figure 2.

**FIGURE 3:**
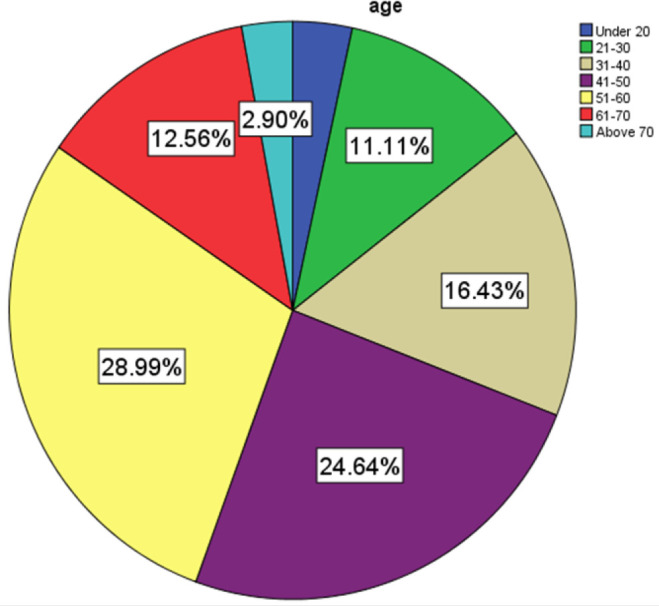
Age Distribution of Patients who had Thyroidectomy Across the Study Period

The age group 51 to 60 years had most of cases at 28.99% followed by 41–50 years at 24.64% as per Figure 3.

**FIGURE 4:**
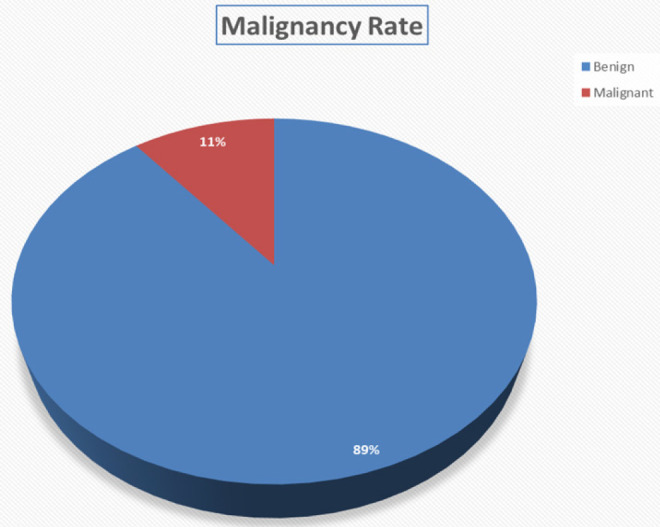
Proportion of Malignancies Among Thyroid Specimens Evaluated Over the Study Period

Eleven percent of case turned out malignant on histopathological evaluation and 89% were benign lesions (Figure 4).

**FIGURE 5:**
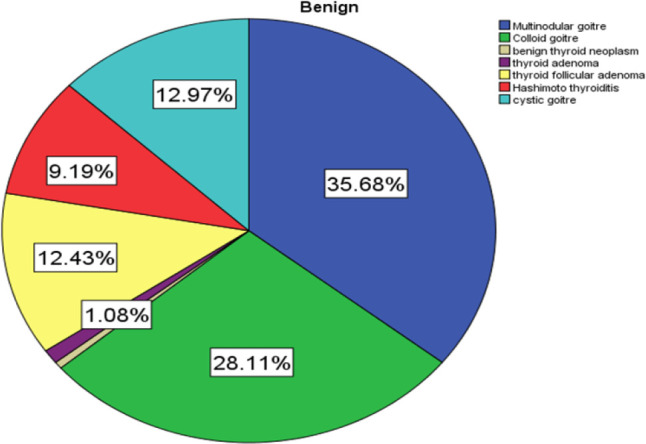
Histological Distribution of Benign Thyroid Lesions

Of the benign thyroid lesions 35.68% were multinodular goiter followed by colloid goiter at 28.11%,%, the third most frequent lesions were cystic goiters at 12.97%, Figure 5.

**FIGURE 6:**
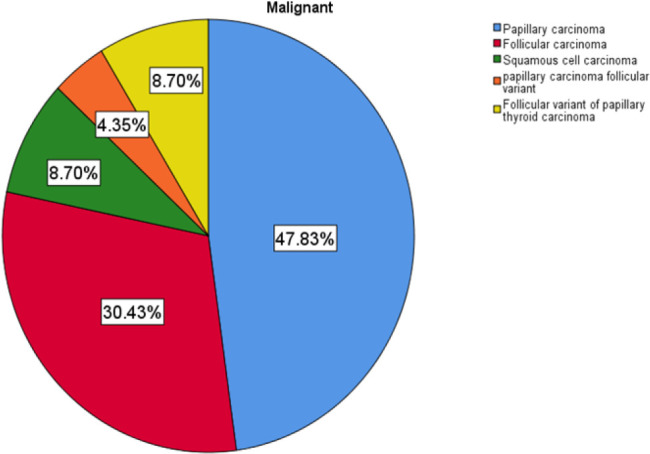
Histological Distribution of Malignant Thyroid Lesions

Of the malignant lesions 47.83% were papillary carcinoma, carcinoma, 30.43% follicular carcinoma and the rest were squamous cell carcinoma 8.7%, follicular variant of papillary thyroid carcinoma 8.7% and 4.35% papillary carcinoma follicular variant.variant as shown in Figure 6.

The correlation between Bethesda category and histological findings revealed a high proportion of malignancy in categories IV and V at 20% and 71.4% respectively. Bethesda category II had 6.5% malignancy proportion whilst category III had 13.9%.

**FIGURE 7:**
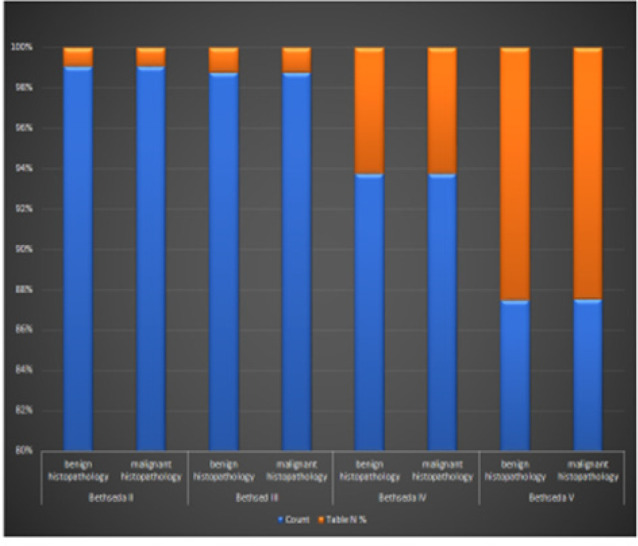
Correlation of Cytological and Histological Findings of Thyroid Tumours

There were no cytological findings reported as Bethesda category I and IV. IV (Figure 7).

Computing sensitivity, sensitivity, specificity and accuracy;

- Sensitivity = TP/(TP+FN) x 100%; 8/(8+14) x 100% = 36.36%
- Specificity = TN=TN/(TN+FP) x 100%; 170/(170+15) x 100%=91.89%
- PPV=TP/(TP+FP) x 100%; 8/(8+15) x100% = 34.78%
- NPV=TN/(TN+FN) x 100%; 170/ (170+14) x 100% = 92.39%
- False positive rate = FP/(FN+TN) x 100%; 15/(15+170) x 100% = 8.10%
- False negative rate = FN/(FN +TP) x 100%; 14/ (14+8) x 100% = 63.64%
- Accuracy = (TP+TN)/(TP+TN+FP+FN) x 100%; (8+170) / (8+170+15+14) x 100% = 85.99%.

## DISCUSSION

The main objective of the study was to obtain determine the sensitivity, specificity and accuracy of FNAC in detecting malignancy for thyroid tumours.

Estimated sample size from the protocol was 372 that on conducting the study a total of 217 participants were recorded and evaluated. Likely explanation for the shortage on sample size target being the limitations on number of elective surgeries which came with the Covid 19 pandemic restrictions during the study period.

Of the 217 cases 10 were deemed unfit for study due to data incompleteness and were excluded from analysis.

A total of 207 subjected had the information regarding gender, age, cytological reports on FNAC and histological findings after thyroidectomy collected and analyzed.

The age of participants ranged from 8 to 81 years, median (IQR) 49 (37–56) years lying within most of published literature.^18,23,28,29^

Of the 207 subjects 185 were females and 22 males, constituting 89.37% and 10.63 %, respectively.

Female to male (F:M) ratio was 8.4:1 connoting female preponderance in keeping with literature. The fact that women are usually more frequent visitors to healthcare centers and have medical follow-up for example during pregnancy plus the influence of risk factors such as hormones and lifestyle-negative factors.

The age group 51–60 years formed a great part of the population with thyroid disease atdisease at 28.99% followed by the 41 to 50 years age group at 24.64% lying within limits of published literature.

The malignancy rate was found to be 11% during the study period matching much of findings by other researchers.^23^

During the study period the Bethesda categories I and VI had no cytological specimens which were slotted in, probably due to ineptitude on part of reporting practices and sample inadequacy, meaninginadequacy, meaning only categories II. III, III, IV and V were evaluated.

The benign lesions had were 35.68%, multinodular goiters, 28.11%, and colloid goiters and 12.97% findings that were in keeping with reported literatures in the region. The dominance of nodular disease and cystic lesions may partly be explained by endemicity of goitrous lesions in the region.

The malignant lesions were predominantly papillary carcinoma 47.83%, follicular carcinoma 30.43% and the rest were squamous cell carcinoma 8.7%%, follicular variant of papillary thyroid carcinoma 8.7% and 4.35% papillary carcinoma follicular variant. The picture depicted throughout the study revealed a predominant papillary histopathology in keeping with most of published literatures^26,27,29^, largely explained by the increasing rollout of iodation programs.

Bethesda category II had a total of 107 amongst which 7 were reported malignant on histopathology forming a 6.5% malignancy rate, category III had 79 cases of which 11 were malignant with a malignancy rate of 13.9%, category IV had 15 cases, 3 were malignant forming a 20% malignancy rate and category V had a total of 7 cases, whereas 5 were malignant with a malignancy rate 71.4%. The risk likelihoods per Bethesda category for categories III, IV and V are within the stated estimates in the literature with exception of category II which at 6.5% that is a way out of the 0–3% risk that is stated.^17,23,28^ The discordance for the observed overshoot in category II can partly be explained by the fuzziness in allotting the categories and the dominantly nodular and cystic lesions which have diagnostic challenges specially sampling, adequacy and mixed pathologies.^4,12,13,16,18–20,23,28–30^

The study had a sensitivity of 36.36%, specificity 91.89%, positive predictive value of 34.78% negative predictive value 92.39% false positive rate 8.10%, false negative rate 63.64% and an overall accuracy of 85.99%.

The dismally low sensitivity may be attributed to the excessive malignant rate in the benign category which were as a consequence left out of reckoning and to the fact that our study took records of cytological reports as they were reported back in time. There were no efforts to retrieve and reread the slides contrary to other studies on the subject^23,27^ and the largely nodular and cystic thyroid lesions in the population which are notorious in diagnostic performances due the overlap in cellular characteristics and sampling adequacy.^13,27,30^ Alluding to an unconvincing likelihoods for picking up the at risk individuals who present with thyroid lesions with malignancy potential for due intervention.

Thyroid cancer is an uncommon disease and as such specificity is the most desirable screening test in the setting as our study had a 91.89% specificity.^29^ The negative predictive value in this study was 93.9% meaning that once FNAC tests a patient as having a benign lesion, then chances of having thyroid malignancy are negligible.

Most of thyroid lesions were benign (89% with a malignancy rate at 11% in keeping with regional rates that contrasts with European and North American rates at about 5%.^23,26,29^ Differences The differences are purportedly related to long standing multinodular goiters in areas endemic for iodine deficiency with higher tendencies for malignant transformation.

Overall diagnostic accuracy for FNAC in evaluating the thyroid lesions stands at 85.99 % which is within the confines of published literature.^23,27,29^

## CONCLUSION

This study reveals high specificity and a low sensitivity for FNAC at detecting malignancy in thyroid nodules. The findings are a baseline data on performance of FNAC in thyroid tumours evaluation at our facility. There is a consistent trend toward papillary thyroid carcinoma (PTC) than follicular thyroid carcinoma (FTC) acrossFT across regions.

### Study Limitations

Lack There was lack of blinding of the histopathologists to the corresponding cytological diagnoses leading up to random error(s) due to the retrospective nature of study with potential for misclassification. The employment of convenience sampling technique may have inadvertently introduced selection bias. There was failure at achieving the estimated sample size within period of study period relative to initial estimation. Single center study limits generalizability. We experienced shoddy record keeping that led to incompleteness of data for enrolment.

### Recommendations

Appraisal of Theof the Bethesda System for Reporting Thyroid Cytopathology (BSRTC) as a standard tool for assigning cytological findings for thyroid tumours.

Empowering the diagnostic performance at Pathology Department by enhancing supportive tools for improved diagnostic yields like sonography, radionuclide studies.

Investing in human resources with the aim of having dedicated team of experts attendant to endocrine lesions, thyroid in particular.

More robust, preferably prospective multi centered studies to work on gaps and lapses pertaining the subject and help validate findings.
